# Supplementary material for: The Unusual Architecture of RNA-Dependent RNA Polymerase (RdRp)’s Catalytic Chamber Provides a Potential Strategy for Combination Therapy against COVID-19
Source: Molecules. 2023 Mar 20;28(6):2806. doi: 10.3390/molecules28062806 (PMC10057333; doi:10.3390/molecules28062806)
Supplement: Supplementary file 1 [file molecules-28-02806-s001.zip › molecules-2266138-supplementary.pdf]

Supplementry Information

The Unusual Architecture of RNA-Dependent RNA Polymerase (RdRp)'s Catalytic Chamber Provides a Potential Strategy for Combination Therapy against COVID-19

Kamel Metwally[1,2*], Nader E. Abo-Dya[1,3], Mohamed Issa Alahmdi[4], Maha Z. Albalawi[5], Galal Yahya[6], Aimen Aljoundi[7], Elliasu Y. Salifu[7], Ghazi Elamin[7], Mahmoud Ibrahim[7,8], Yasien Sayed[9], Sylvia Fanucchi[9] and Mahmoud E.S Soliman[7*]

[1]Department of Pharmaceutical Chemistry, Faculty of Pharmacy, University of Tabuk, Tabuk 71491, Saudi Arabia

[2]Department of Pharmaceutical Medicinal Chemistry, Faculty of Pharmacy, Zagazig University, Zagazig 44519, Egypt

[3]Department of Pharmaceutical Organic Chemistry, Faculty of Pharmacy, Zagazig University, Zagazig 44519, Egypt

[4]Department of Chemistry, Faculty of Science, University of Tabuk, Tabuk 71491, Saudi Arabia

[5]Pharm D Program, Faculty of Pharmacy, University of Tabuk, Tabuk 71491, Saudi Arabia

[6]Department of Microbiology and Immunology, Faculty of Pharmacy, Zagazig University, Zagazig 44519, Egypt

[7]Molecular Bio-computation and Drug Design Laboratory, School of Health Sciences, University of KwaZulu-Natal, Westville Campus, Durban 4001, South Africa

[8]CompChem Lab, Chemistry Department, Faculty of Science, Minia University, Minia 61519, Egypt.

[9]Protein Structure-Function Research Unit, School of Molecular and Cell Biology, University of the Witwatersrand, Johannesburg, 2050, South Africa

*Corresponding Author: Kamel Metwally, Email: kametwally@ut.edu.sa

& Mahmoud E.S. Soliman, Email: soliman@ukzn.ac.za

Website: http://soliman.ukzn.ac.za

Telephone: +27 (0) 31 260 8048, Fax: +27 (0) 31 260 78

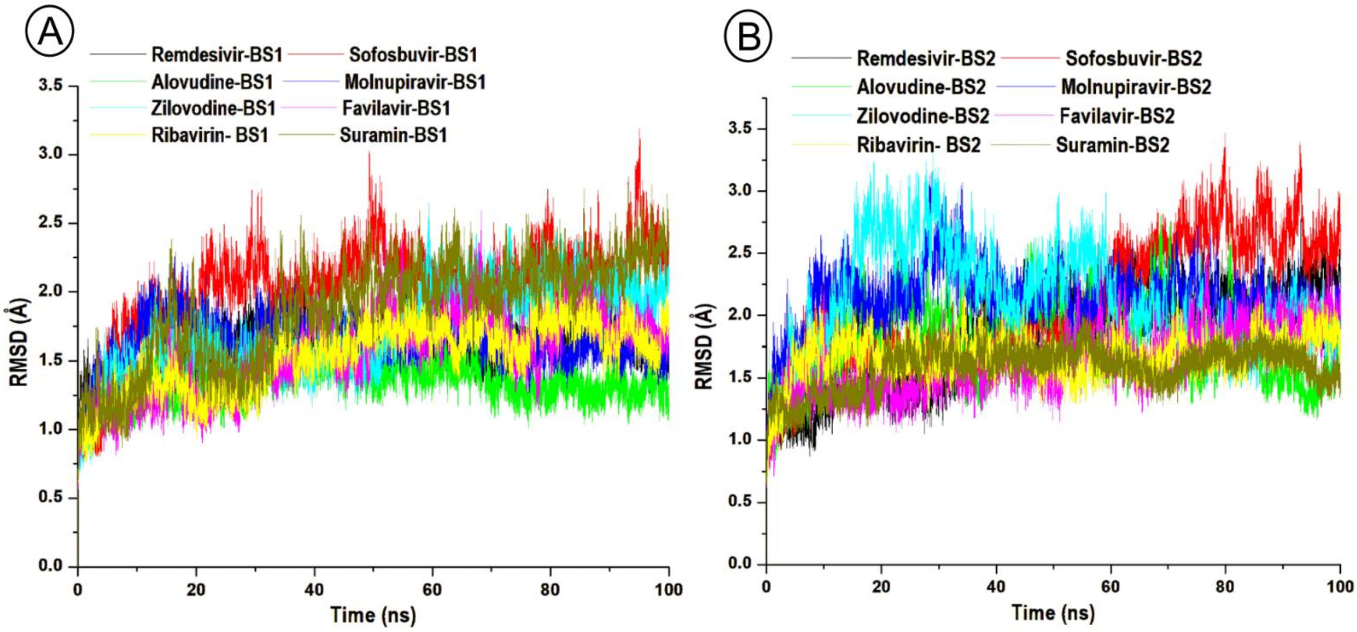

Figure S1: Structural representation of alterations occurring during the binding of the selected eight antiviral to RdRp Catalytic Chamber BS1 and BS2. (**A**) The conformational stability, C-α atoms RMSD of the eight antiviral to RdRp Catalytic Chamber BS1 with the different colors showed. (**B**) Conformational stability, C-α atoms RMSD of the eight antiviral inhibitors to RdRp Catalytic Chamber BS2 ran for 100 ns MD simulation.

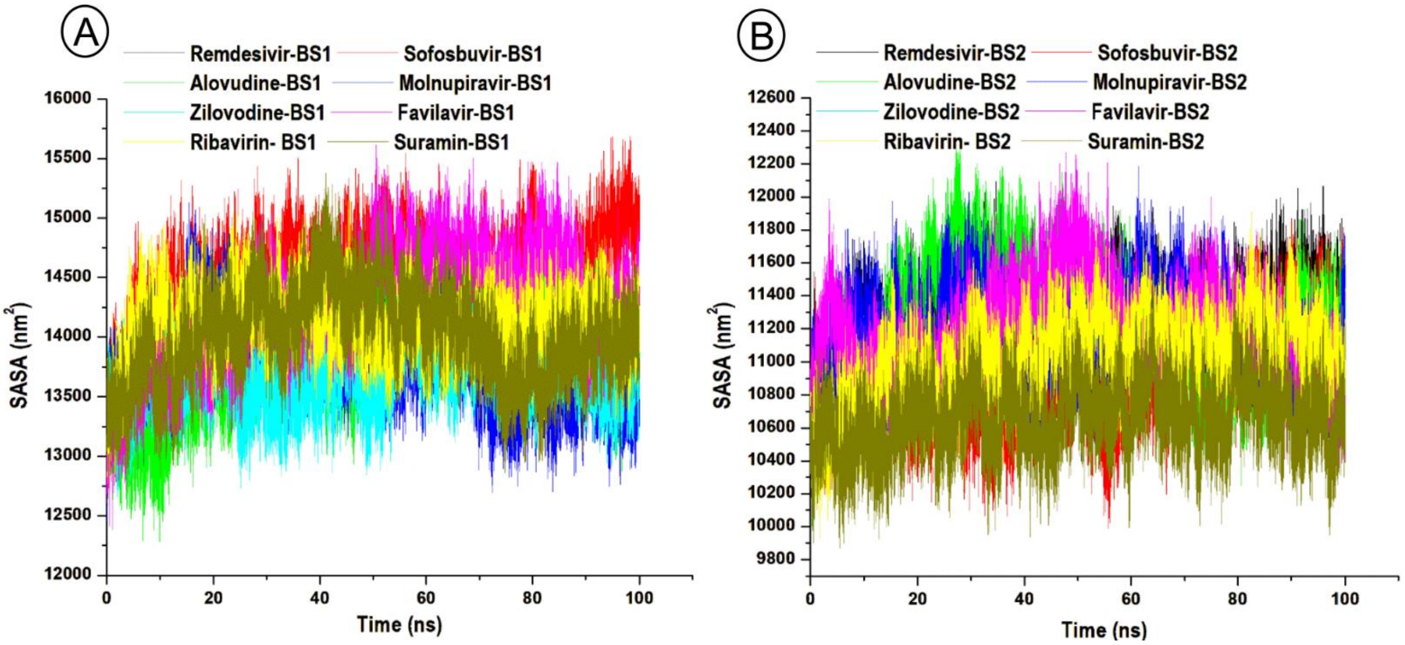

Figure S2: Structural representation of alterations occurring during the binding of the selected eight antiviral to RdRp Catalytic Chamber BS1 and BS2. The (**A**) Solvent accessible surface area **(**SASA**)** for the antiviral inhibitors at BS1. (**B**) Solvent accessible surface area **(**SASA**)** for the antiviral inhibitors at BS2 Catalytic Chamber.

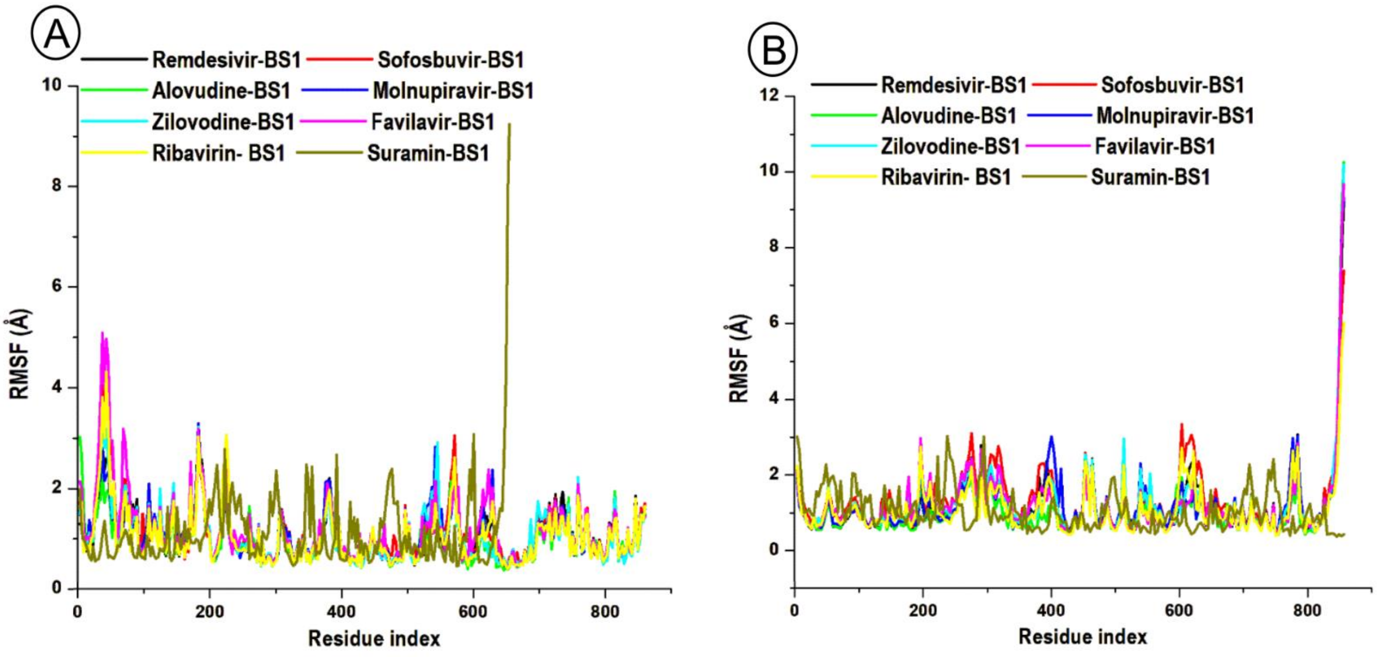

Figure S3: The time evolution RMSF of each residue of the enzyme C-α atom over 100 ns for **(A)** the selected eight antiviral inhibitors bind to RdRp Catalytic Chamber BS1, **(B)** the selected antiviral inhibitors bind to RdRp Catalytic Chamber BS2 respectively.
